# AAV-ie-K558R mediated cochlear gene therapy and hair cell regeneration

**DOI:** 10.1038/s41392-022-00938-8

**Published:** 2022-04-22

**Authors:** Yong Tao, Xiaoyi Liu, Liu Yang, Cenfeng Chu, Fangzhi Tan, Zehua Yu, Junzi Ke, Xiang Li, Xiaofei Zheng, Xingle Zhao, Jieyu Qi, Chao-Po Lin, Renjie Chai, Guisheng Zhong, Hao Wu

**Affiliations:** 1 Department of Otolaryngology-Head and Neck Surgery, Shanghai Ninth People’s Hospital, Shanghai Jiao Tong University School of Medicine, Shanghai, 200011 PR China; 2 Ear Institute, Shanghai Jiao Tong University School of Medicine, Shanghai, 200011 PR China; 3 Shanghai Key Laboratory of Translational Medicine on Ear and Nose Diseases, Shanghai, 200011 PR China; 4 iHuman Institute, ShanghaiTech University, Shanghai, 201210 PR China; 5 School of Life Science and Technology, ShanghaiTech University, Shanghai, 201210 PR China; 6 State Key Laboratory of Bioelectronics, Department of Otolaryngology Head and Neck Surgery, Zhongda Hospital, School of Life Sciences and Technology, Jiangsu Province High-Tech Key Laboratory for Bio-Medical Research, Southeast University, Nanjing, 210096 PR China; 7 Co-Innovation Center of Neuroregeneration, Nantong University, Nantong, 226001 PR China; 8 Institute for Stem Cell and Regeneration, Chinese Academy of Science, Beijing, PR China; 9 Beijing Key Laboratory of Neural Regeneration and Repair, Capital Medical University, 100069 Beijing, PR China

**Keywords:** Gene therapy, Auditory system

## Abstract

The cochlea consists of multiple types of cells, including hair cells, supporting cells and spiral ganglion neurons, and is responsible for converting mechanical forces into electric signals that enable hearing. Genetic and environmental factors can result in dysfunctions of cochlear and auditory systems. In recent years, gene therapy has emerged as a promising treatment in animal deafness models. One major challenge of the gene therapy for deafness is to effectively deliver genes to specific cells of cochleae. Here, we screened and identified an AAV-ie mutant, AAV-ie-K558R, that transduces hair cells and supporting cells in the cochleae of neonatal mice with high efficiency. AAV-ie-K558R is a safe vector with no obvious deficits in the hearing system. We found that AAV-ie-K558R can partially restore the hearing loss in *Prestin* KO mice and, importantly, deliver *Atoh1* into cochlear supporting cells to generate hair cell-like cells. Our results demonstrate the clinical potential of AAV-ie-K558R for treating the hearing loss caused by hair cell death.

## Introduction

Cells of the cochlea, such as hair cells (HCs) and supporting cells (SCs), are essential for hearing.^1^ While sensorineural hearing loss can result from genetic mutations in both HCs and SCs, non-genetic stresses, such as noise, ototoxic medicines, or aging, can also induce deafness through damaging HCs. In either case, these damages are irreversible in mammals who do not have the ability to regenerate cochlear cells. While current treatments, such as hearing aids and cochlear implants, can alleviate the hearing loss in some patients, these approaches are limited by their sensitivity and perception of natural sounds in noisy environments.

HCs in cochleae consist of two different types: outer HCs (OHCs), which amplify sound, and inner HCs (IHCs), which directly convert mechanical forces to electrical signals.^1^ HCs anchor the sensory epithelium to the basilar membrane which is essential for maintaining the environment for proper HC function. Notably, since SCs have the potential to transdifferentiate into HC-like cells,^2,3^ HC regeneration is considered as a potential treatment for acquired deafness caused by non-genetic stresses.^4–6^

Gene therapies have emerged as important treatments for genetic diseases.^7–12^ Indeed, current progresses also demonstrate their potential for treating hearing loss. Several genes, such as *tmc1*, *clrn*, and *otof*, when being delivered to cochleae, can restore hearing function in animal models.^7,10,13,14^ A previous study reported that HC regeneration in adult cochleae can restore auditory function in deafness guinea pig models,^15^ though further studies are required to extend this finding to other animal models.

Adeno-associated viruses (AAVs) have been shown to possess high safety in both animal models and humans and are widely used to deliver genetic materials to cells for gene therapies in many different organs and diseases.^9,16–25^ In the hearing field, earlier studies have found that Anc80L65 is a promising vector for delivering harmonin to treat deafness caused by HC dysfunction.^16^ However, its efficiency to transduce HCs remains to be improved. Previously, we developed a synthetic AAV, AAV-ie, which targets SCs and HCs.^22^ AAV-ie can regenerate HC-like cells through delivering the transcription factor, *Atoh1*, which transdifferentiates SCs into HC-like cells.^2,3,22,26^ However, its targeting efficiencies for SCs or HCs need to be improved, especially in the basal region of cochleae.

In the present study, we performed mutational screening on AAV-ie capsid. We generated a repertoire of mutants on the amino acid sequence of AAV-ie capsids to manipulate phosphorylation/ubiquitination of AAVs in cells. We demonstrated that a particular amino acid-mutant AAV-ie capsid, AAV-ie-K558R, can transduce SCs with high efficiency and is suitable for correcting dysfunctional genetic mutations or for HC-like regeneration.

## Results

### Mutations of surface amino acids of capsids improve the cochlear transduction efficiency of AAV-ie in vivo

**Fig. 1:**
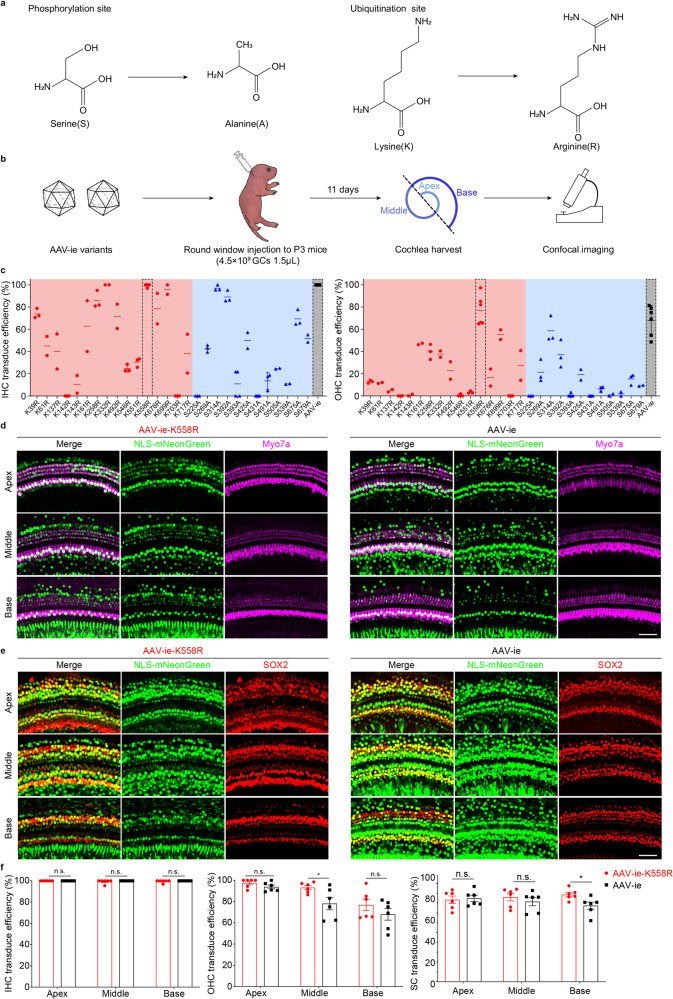
Capsid amino acid mutation increased AAV transduction. **a** Amino acid mutation strategy. For the phosphorylation site, serine(S) was substituted with alanine(A), and for the ubiquitination site, lysine(K) was substituted with arginine(R). The most structurally similar amino acids were chosen to prevent any significant property change to the capsid while preventing phosphorylation/ubiquitination modifications. **b** Schematic of AAV mutant vector screening. P3 mice were injected with AAV-ie mutants via round window injection at a dose of 4.5 × 10^9^ genome-containing (particles) (GCs) per ear, and the cochleae were harvested 11 days after virus injection. The cochleae were dissected into three parts indicated by dashed line, representing low (apex), middle (middle) and high (base) frequencies. Then cochleae were fixed and immunohistochemically labeled, followed by confocal imaging. **c** Transduction efficiencies of AAV-ie and AAV-ie-S/K mutant vectors for outer and inner hair cells at an equal dose (4.5 × 10^9^ GCs). AAV-ie-K558R shows comparable transduce efficiency with AAV-ie. **d**, **e** Comparison of AAV-ie and AAV-ie-K558R transduction efficiency. Imaged for virally expressed NLS-mNeonGreen (green) and stained for Myo7a(magenta), SOX2(red). Given that although both AAV-ie and AAV-ie-K558R can efficiently transduce hair cells and supporting cells throughout the cochlea, AAV-ie-K558R shows the same or even higher transduce efficiency in part of the cochlea. Scale bar: 50 μm. **f** Statistics analysis of AAV-ie and AAV-ie-K558R transduce efficiency. Data shown here as mean ± SEM. Significance tests were performed between AAV-ie and AAV-ie-K558R. *p* Value is calculated by Student’s *t* test. *p < 0.05, **p < 0.01, and ***p < 0.001

The protein ubiquitination is associated with AAV transduction efficiency and blocking ubiquitination can increase AAV transduction efficiency in vitro and in vivo.^27,28^ Our previous study showed that AAV-ie can transduce cochlear cells in both rodents and humans. However, the transduction efficiency of AAV-ie is low in the basal region of cochleae. We reasoned that mutating capsid amino acids on the exposed side of AAV-ie may increase the transducing efficiency^29–31^ (Fig. 1a). To test this hypothesis, we generated 28 AAV-ie variants with different mutations on the capsid sequence (Supplementary Table 1). To increase the screening accuracy, we performed in vivo screening of these AAV-ie variants by round window membrane (RWM) injection in the P3 cochleae of neonatal mice with equal dose (4.5 × 10^9^ genome-containing particles, GCs) per ear (Fig. 1b). The cochleae were harvested for immunostaining at P14. Myo7a antibody was used to label hair cells and nuclear localization signal (NLS)-mNeonGreen signal indicated the nuclei localization of the transduced cells in the cochlea.

The transduction efficiencies of the mutated vectors were analyzed and compared with the AAV-ie in animals under identical conditions. As shown in Fig. 1c, while most AAV-ie variants did not show any increased transduction efficiency in the basal region of the cochlear (Supplementary Fig. 1), some even has decreased instead. One of these variants, AAV-ie-K558R, stood out with high transduction efficiency in both OHCs and IHCs in the basal region of cochleae. Further comparison of the transduction profile of AAV-ie-K558R and AAV-ie shows that AAV-ie-K558R have increased transduce efficiency both in the OHCs in the middle region of cochlea and the SCs in the basal region (Fig. 1d–f). We assessed the targeting efficiency of AAV-ie-K558R in the vestibular system which is responsible for detecting linear motion and sensing gravity. We observed NLS-mNeonGreen expression throughout the sensory epithelium of the mouse utricle and nearly 100% transduction efficiency of AAV-ie-K558R in HCs and SCs (Supplementary Fig. 2).

Together, these experiments demonstrated that AAV-ie-K558R can efficiently target different types of cells in cochleae and utricles, revealing their potential as suitable vector to mediate gene correction for auditory diseases or to regenerate HC-like cells in the cochlear or vestibular system.

### AAV-ie-K558R is a safe vector

**Fig. 2:**
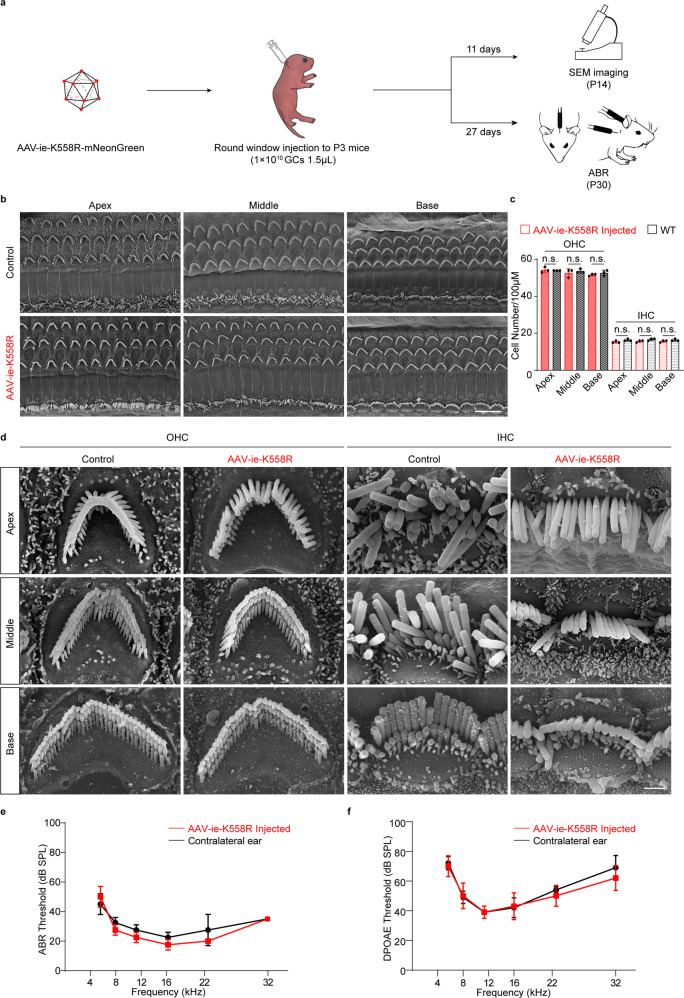
AAV-ie-K558R is a safe vector. **a** Schematic experimental setup of AAV-ie-K558R study to investigate the vector safety. **b** Representative SEM images of WT control and AAV-ie-K558R-NLS-mNeonGreen-injected cochlea at apical, middle and basal region. Scale bar: 10 μm. **c** OHCs and IHCs numbers per 100 μm of uninjected WT mice and AAV-ie-K558R-NLS-mNeonGreen-injected cochlea at apical, middle and basal region. *n* = 3 mice. *p* Value is calculated by Student’s *t* test. n.s. refer to no significance. **d** Magnification SEM images of outer hair cells (OHCs) and inner hair cells (IHCs) of P14 WT control and AAV-ie K558R-NLS-mNeonGreen-injected cochlea at apical, middle, and basal regions. Scale bar:1 μm. **e**, **f** ABR and DPOAE thresholds of AAV-ie-K558R-NLS-mNeonGreen injected ear and un-injected contralateral ear on day 27 after virus injection at P3. Virus injection and the AAV vector show no effects on mice hearing

An earlier study suggested that AAV-ie is safe in mouse inner ear and did not exhibit any toxic effect on HCs or the auditory function.^22^ We tested whether AAV-ie-K558R has a similarly safe profile as AAV-ie. We injected AAV-ie-K558R (1 × 10^10^ GC) per ear through the RWM and collected cochleae for scanning electron microscopy (SEM) analyses. The SEM results indicated that HCs underwent no or little morphological change after injection with properly oriented hair bundles (Fig. 2b, d). We counted the HC numbers in representative fields of view and observed no difference between AAV-injected ears and controls, indicating no significant hair cell loss (Fig. 2c). We then tested whether AAV-ie-K558R injection affects hearing ability by testing auditory brain-stem responses (ABRs) which reflect the auditory function of live animals and distortion product otoacoustic emissions (DPOAE), which reflect the outer hair cell integrity. Again, the thresholds exhibited no differences between injected ears and controls (Fig. 2e, f).

We next tested whether AAV-ie-K558R had any unexpected effects on animal behaviors which indicate gross vestibular dysfunctions, such as circling behavior or other gait abnormalities. By four weeks after virus injection, we did not observe any abnormalities of gait or circling behavior in open–field pathway trace test. Narrow beam walking tests were further examined and no obvious differences was observed between virus injected mice and uninjected control mice (Supplementary Fig. 3). With these results, we conclude that AAV-ie-K558R exhibit no negative effects on the auditory and vestibular system.

### AAV-ie-K558R-*Prestin* partially reverses deafness in *Prestin* KO mice

**Fig. 3:**
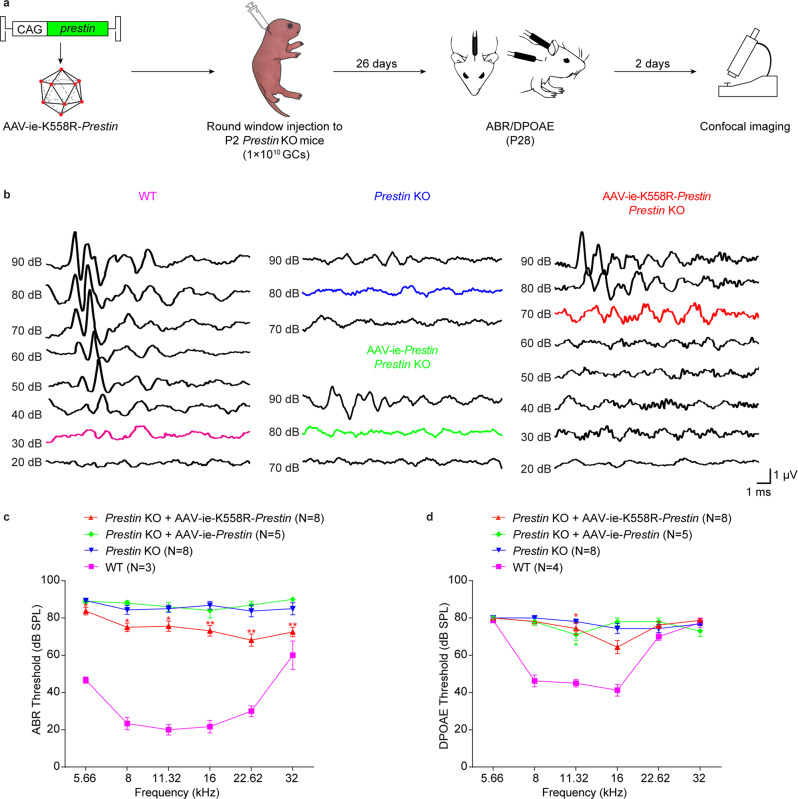
AAV-ie-K558R-*Prestin* restores auditory function of *Prestin* KO mice. **a** Schematic of *Prestin* KO mice hearing recovery experiments. AAV-ie and AAV-ie-K558R vectors were used to package a single strand genome that expresses Prestin driven by the CAG promoter. **b** Families of ABR waveforms recorded at P28 from uninjected WT mouse, *Prestin* KO mouse without treatment and *Prestin* KO mouse injected with AAV-ie-*Prestin* or AAV-ie-K558R-*Prestin*. ABRs were recorded using 16 kHz tone bursts at increasing sound pressure levels in 10 dB steps. Thresholds were determined by the presence of Peak 1 and are indicated by colored traces. Scale bar applies to all families. **c**, **d** ABR and DPOAE thresholds plotted as a function of stimulus frequencies for *Prestin* KO mice injected with AAV-ie-*Prestin* (green) or AAV-ie-K558R-*Prestin* (red). Uninjected contralateral ear of *Prestin* KO mice were used for negative control (blue) and uninjected WT mice were used as positive control (purple). Data were shown as Mean ± SEM. Significance (*P < 0.05, **P < 0.01, ***P < 0.001) was calculated using multiple *t* test between AAV-injected group and contralateral uninjected group

It is challenging to perform the gene therapy in deafness models caused by gene deficit in OHCs due to the lack of available AAVs that can transduce OHCs with high efficiency. To test whether AAV-ie-K558R has the potential to restore hearing loss caused by HC gene dysfunction, we employed *Prestin* KO mice in which the Prestin protein was ablated in OHCs.^32^ Prestin is a critical molecule that specifically locates in OHCs and plays a crucial role in mediating their electromotility.^33–36^ In *Prestin* KO mice, OHCs do not possess the non-linear membrane capacity and thus the hearing threshold was significantly higher than the wild-type (WT) (Fig. 3a, b). The *Prestin* KO mice did not show utricle deficits or abnormalities of circling or gait behavior (data not shown).

We next generated the AAV-ie-K558R-*Prestin* and injected this virus into *Prestin* KO mice at P1-2 to examine whether it has the potential to treat genetic auditory disease caused by gene dysfunction in OHCs. To test whether the AAV-mediated Prestin overexpression in HCs can restore hearing function in the *Prestin* KO mice, ABR experiments were performed in different groups of mice. We measured the ABR threshold of mice 1 month after virus injection at P1-2 and observed the partial recovery of ABR with the more pronounced effect in the high frequency range (Supplementary Fig. 4; Fig. 3b, c), and DPOAE result suggest that AAV-ie-K558R-*Prestin* have 10 dB rescue in 16 kHz. Next, we tested the expression of Prestin by harvesting the cochleae two months after virus injection and examine the expression of Prestin in hair cells from *Prestin* KO mice. We observed substantial expression of Prestin in HCs of *Prestin* KO mice two months after virus injection (Supplementary Fig. 5). We also packaged *Prestin* into AAV-ie vector and injected into neonatal *prestin* KO mice, but we didn’t observe any ABR threshold improvement or sufficient Prestin expression in OHCs 1 month after injection (Fig. 3c, d). Together, our results suggest that AAV-ie-K558R can be used as a potential vector to deliver genes to OHCs and for gene therapy in deafness mouse models with hair cell deficits.

### In vivo HC-like cell regeneration mediated by AAV-ie-K558R-*Atoh1*

**Fig. 4:**
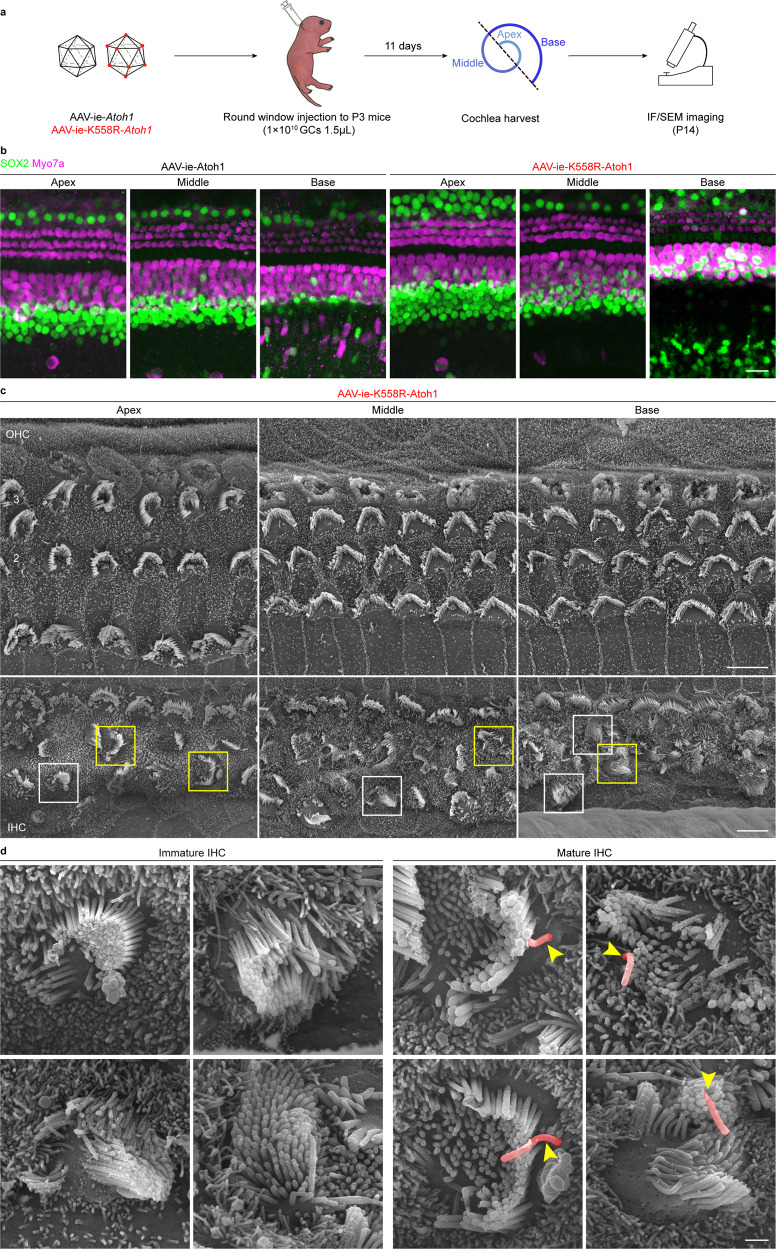
AAV-ie-K558R-*Atoh1* can regenerate HC-like cells in neonatal mice. **a** Schematic of AAV-induced hair cell regeneration in WT C57BL/6 neonatal mice. **b** Immunofluorescence imaging of cochlea transduced with AAV-ie-*Atoh1* and AAV-ie-K558R-*Atoh1* at dose of 1 × 10^10^ GCs. Scale bar: 20 μm. **c** SEM images of a cochlea injected with AAV-ie-K558R-*Atoh1* on P14 in the apical, middle and basal regions. Top row, three rows of OHCs are numbered. Bottom row, boxes show immature (white) and mature (yellow) HC-like cells regenerated by AAV-ie-K558R-*Atoh1*. Scale bar: 5 μm. **d** Magnified SEM images of the immature and mature regenerated HC-like cells shown in **c** as numbered. Stereocilium were observed in these regenerated cells, and kinocilium was artificially colored as red. Immature regenerative cells did not show kinocilia and obvious polarity, whereas those regenerative cells that grew kinocilium (yellow arrowhead) with certain polarity were considered more mature. Scale bar: 1 μm

It is thought that HC regeneration may help restoring hearing losses caused by aging, noise or ototoxic medicine.^1^ The transcription factor Atoh1 was demonstrated to be able to transdifferentiate SCs into HCs.^4,5^ Our earlier study showed that AAV-ie can deliver *Atoh1* to SCs and transdifferentiate them into HC-like cells.^22^ To assess the potential of the AAV-ie-K558R vector for HC regeneration, we used AAV-ie-K558R-*Atoh1* (AAV-ie-K558R-*Atoh1*) to deliver mouse *Atoh1* into neonatal mouse cochleae in vivo (Fig. 4a). AAV-ie-K558R-*Atoh1* viruses (1 × 10^10^ GCs per ear) were injected into the cochleae via RWM at P3 and the cochleae were harvested at P14. In the AAV-ie-*Atoh1* injection group, some new HC-like cells expressing Myo7a were presented in the sensory region (Fig. 4b). In the AAV-ie-K558R-*Atoh1* injection group, we observed a large number of new HCs expressing Myo7a in the greater epithelial ridge (GER) region. Notably, some new HC-like cells maintained the expression of *Sox2*, implying that these newly regenerated cells may be at different developmental stages (Fig. 4b). We observed little difference in the number of new HCs in the AAV-ie-K558R-*Atoh1* group as compared to other *Atoh1* overexpression approaches, such as the genetic method used in previous studies,^5,37–39^ suggesting that AAV-ie-K558R is a highly efficient viral vector for HC regeneration.

Finally, we used SEM to quantitate the morphology of newly regenerated cells. AAV-ie-K558R-*Atoh1* caused robust HC-like regeneration with hair bundles grown from the HC-like cells below the IHC region (Fig. 4c). Consistent with our earlier study,^22^ AAV-ie-*Atoh1* can also induce HC-like cell regeneration (Supplementary Fig. 6). Yet, AAV-ie-K558R-*Atoh1* has the ability to generate some HC-like cells in the GER region as demonstrated by SEM (Supplementary Fig. 7). We observed the different morphology of newly generated HC-like cells, with cells developing a kinocilium, representing a more mature developmental stage of hair cells in cochleae (Fig. 4d). Together, these results show that AAV-ie-K558R could robustly induce HC-like cell regeneration in cochleae by delivering *Atoh1*.

## Discussion

In this study, we optimized AAV-ie by changing the presumably exposed amino acids of its capsids, reducing ubiquitination or phosphorylation. Our results showed that the newly generated AAV variant, AAV-ie-K558R, seems to be safe as well as beneficial for both hair cell regeneration and gene therapies. Most AAV-ie variants did not exhibit higher targeting efficiency, but in contrast had a lower targeting efficiency, suggesting that reduced ubiquitination or phosphorylation doesn’t necessarily lead to higher transduction efficiency. Therefore, the exact mechanism underlying the transduction efficiency requires further studies. These results also reveal the importance of performing screening experiments in order to determine the optimal AAV for different tissues. Nonetheless, our results advise that peptide insertion and amino acid mutations are feasible for creating appropriate AAVs to effectively transduce the cells not only in cochleae, but also in other tissues.

Our finding that AAV-ie-K558R partially restored hearing function in *Prestin* KO mice represents a substantial advance in the field of gene therapies targeting auditory diseases, since the effective delivery of genes into OHCs has always been a significant challenge.^16,40^ In this study, we achieved only partial restoration of the auditory function with AAV-ie-K558R. Several factors might explain this partial restoration phenotype. First, AAV-ie-K558R can transduce the IHCs as well, and overexpression of Prestin in IHCs may cause dysfunction and thus prevent the full recovery of auditory function. Second, the expression level of Prestin in OHCs may have to be precisely controlled in order to fully regain the function of OHCs. In this study, we used a widely used gene regulation promotor, CAG, which may not be the optimal choice to control the expression of Prestin in OHCs. Further studies are required to discover the optimal gene regulation elements in the AAV delivery approach for the cochleae. It will be important to develop a modulatory way to control gene expression with AAVs in cochleae and in other tissues as well.

Earlier studies used several viral vectors, including lentiviruses and adenoviruses, to deliver *Atoh1* into the cochlea, and the *Atoh1* overexpression was reported to cause the regeneration of HC-like cells and even partially restored hearing in deaf guinea pigs.^3,4,15,38,39,41,42^ The FDA approved a human clinical trial using an adenovirus vector to deliver human *Atoh1* gene into the cochlea (NCT02132130), however, the efficiency and effectiveness of adenovirus-mediated HC regeneration needed to be improved. Here, we show that AAV-ie-K558R-*Atoh1* can induce many new HCs in the sensory region and the efficiency is comparable with previous genetic mouse work. Considering the regeneration efficiency and strong immune response of adenoviruses, it appears that AAV-ie-K558R has more advantages for HC regeneration. It’s worthy to note the new HC-like cells generated by AAV-ie-K558R were relatively immature. One possible approach would be to deliver *Atoh1* together with other transcription factors in combination, such as *ikzf*, *six1*, etc., to improve the maturity and functionality of newly transdifferentiated cells.

In summary, AAV-ie-K558R is the first reported AAV vector that can be used for gene therapy in the deafness mouse model and can induce HC-like cell regeneration in the neonatal mice. We envision that further development and refinement of AAV-ie-K558R will be crucial for better efficacies of gene therapy or HC cell regeneration in different auditory diseases. Large-scale promoter screens for developing specific AAVs transducing HCs, SCs or SGNs in cochleae will help to design next-generation AAVs. With specific promoters in hand, AAV-ie-K558R-mediated gene therapy may have the potential, not only for recovering hearing function from deafness mouse model caused by genetic dysfunction of HCs or SCs, but may also for alleviating environmental and age-induced deafness.

## Materials and methods

### Animals

Animals were used in accordance with standard ethical guidelines. Both sexes of C57BL/6 mice (Jackson Laboratories) were used in this study at an equal ratio. The number of days since birth was counted from postnatal day 0 (P0). *Prestin* KO mice were kindly provided by Dr. Zhi-yong Liu from CAS Center for Excellence in Brain Science and Intelligence Technology, Shanghai Institutes for Biological Sciences, Chinese Academy of Sciences and then were housed under a 12 h light/dark cycle at a room temperature of 22 ± 1 °C with food and water available ad libitum. The primers used for *Prestin* KO mice genotyping are F1: 5’-CCACCACGTTTAGTAGCATC-3’, R1: 5’-ACTGTGATGAACATGAGCCA-3’, F2: 5’-AGAGCACACCTGCGCTCTTC-3’, R2: 5’-AGTGTGGATGTCAGGCAGAGTA-3’. All experiments were approved by the Institutional Animal Care and Use Committee of ShanghaiTech University, China, and all efforts were made to minimize the number of mice used and their suffering.

### Virus preparation

The C-terminal Flag-tagged NLS-mNeonGreen was cloned into the AAV plasmid containing the cytomegalovirus enhancer/chicken β-actin (CAG) promoter and the woodchuck hepatitis virus post-transcriptional regulatory element (WPRE) cassette, which was flanked by AAV2 inverted terminal repeats. All AAV serotype vectors were produced in HEK 293T cells co-transfected with rep-cap fused plasmid and a helper plasmid. AAVs were purified by iodixanol gradient ultracentrifugation. In brief, cultured medium was collected twice every 48 h after transfection. The cell lysate was treated with chloroform, and the supernatant was collected. The medium and supernatant were combined and concentrated by precipitation with 10% PEG 8000 and 1.0 M NaCl at 4 °C overnight. After centrifugation, the pellet was resuspended in 1 × PBS buffer with Benzonase. 15%, 25%, 40%, and 60% iodixanol solutions were carefully layered and then the generated viral suspension was overlaid, followed by centrifugation at 350,000 × *g* for 90 min at 10 °C. Following ultracentrifugation, the AAV-containing 40% fraction was collected. The buffer was exchanged to remove the iodixanol and concentrate the purified virus. The genome-containing titers of AAVs were determined by SYBR (Roche) analysis using primers targeting the WPRE region. The qPCR primers for WPRE are listed as follows: forward, 5′-GTCAGGCAACGTGGCGTGGTGTG-3′; reverse, 5′-GGCGATGAGTTCCGCCGTGGC-3′.

### Structure analysis of AAV-ie capsid

For amino acid phosphorylation and ubiquitination site prediction, we followed a previously published method.^43^ In brief, phosphorylation sites in AAV-ie capsid were predicted with NetPhosK (http://www.cbs.dtu.dk/services/NetPhosK), Phosida (Phosphorylation Site Database; http://www.phosida.com), KinasePhos (http://kinasephos.mbc.nctu.edu.tw), and Scansite (http://scansite.mit.edu). Ubiquitination sites were predicted with UbiPred (http://iclab.life.nctu.edu.tw/ubipred/) prediction servers.

### Site-directed mutagenesis

Serine (S)→alanine (A) and lysine (K)→arginine (R) mutations were introduced into AAV-ie *rep/cap* plasmid with Phanta Max Super-Fidelity DNA Polymerase (Vazyme, Cat.P505) according to the manufacturer’s protocol. Briefly, a one-step PCR amplification of the target sites was performed for 28 cycles with the primers (Supplementary Table S1). The PCR products were digested by Dpn1 enzyme (NEW ENGLAND Bio Labs, cat. R0176S) for 2 h. Then ten microliters of the recombinant product were transformed into DH5α chemically competent cell (Shanghai Weidi Biotechnology CO., Ltd, cat. DL1001). Plasmids were isolated with TIANprep Mini Plasmid Kit (TIANGEN BIOTECH(BEIJING) CO., LTD, cat.DP118) and verified by DNA sequencing (Applied Biosystems 3730XL genetic analyzer; BioSune, Shanghai, China).

### AAV injection by round window injection

We followed a previously published surgery procedure. Briefly, the neonatal mice were anesthetized by low temperature. P2–3 mice were placed in an ice bath for 2–3 min until loss of consciousness and then removed to an ice pad for subsequent surgical procedures. The surgery was limited to 5–10 min. Surgery was performed only on the right ear of each animal. Upon anesthesia, a post-auricular incision was made to expose the otic bulla and visualize the cochlea. Guided by the relative position between the temporal bone and the facial nerve, the round window was exposed. Special care was taken to avoid damage to the facial nerve during surgery. Injections were performed through the RWM with a glass micropipette (25 μm) controlled by micromanipulator UMP3 UltraMicroPump (World Precision Instruments). The volume of the injected materials was controlled at ~1.5 μL per cochlea within 1 min per AAV. After the injection, the skin incision was closed using veterinary tissue adhesive (Millpledge Ltd, UK). Pups were subsequently returned to the 37 °C warming pad for 10 min and then returned to their mother for continued nursing.

For RWM injection in adult mice, 4-week-old C57/B6 mice were used. Before surgery, the mice were anesthetized with ketamine (100 mg/kg) and xylazine (25 mg/kg). The otic bulla was opened to expose the round window niche. Injections were performed through the RWM with a glass micropipette (25 μm) controlled by a micromanipulator UMP3 UltraMicroPump (World Precision Instruments). The volume of the injected materials was controlled at ~2.0 μL per cochlea within 1 min per AAV. After the injection, the skin incision was closed using veterinary tissue adhesive (Millpledge Ltd, UK).

### Hearing tests via ABR measurements

ABR is a method to assess hearing by measuring the hearing threshold. Based on the Tucker-Davis Technology system III (Tucker-Davies Technologies, TDT, Gainesville, FL, USA), this test closed-field ABR was recorded from mice anesthetized with ketamine (100 mg/kg) and xylazine (25 mg/kg) in a sound-attenuated room, and changes in the electrical activity of the brain in response to sound were recorded via electrodes. The ABR responses were elicited in tone bursts at five frequencies (4, 8, 12, 16, 24, and 32 kHz). The acquired response signal of ABR was amplified (10,000 times), filtered (0.1–3 kHz), averaged, and presented in the computer-based data-acquisition system, BioSigRZ (TDT, Gainesville, FL, USA). The sound level was raised in 5 to 10 dB steps from 0 to 90 dB sound pressure level (decibels SPL). At each level, 1024 responses were averaged (with alternating stimulus polarity) after artifact rejection, using the lowest sound pressure level that elicited a visually detectable response as the rejection threshold.

### Immunofluorescence staining

Injected cochleae were harvest on day 10 after injection. The temporal bone was fixed in 4% paraformaldehyde (36314ES76, Yeasen) for 2 h at room temperature and then decalcified in 0.5 M EDTA (ST066, Beyotime) for 1-2 h at room temperature. Specimens were then cut into apical, middle, basal regions.

For cross-section, cochleae were excised and fixed in 4% PFA for 2 h and then decalcified in 0.5 M EDTA for 2 h at room temperature. Specimens were cryo-protected in 30% sucrose in 1 × PBS (SH30256.FS, Hyclone) overnight at 4 °C and then embedded in tissue freezing medium (14020108926, Leica), frozen, and sectioned (14 μm).

Samples were blocked with 10% donkey serum in 0.3% Triton X-100 dissolved in 1 × PBS at room temperature for 1 h. Then, the samples were stained with antibodies against myosin 7 A (Myo7a, #25-6790 Proteus Biosciences, 1:1000), Sox2 (Sox2, #sc-17320, Santa Cruz Biotechnology, 1:1000), Flag (Flag, #F3165, Sigma-Aldrich, 1:1000), together with corresponding secondary antibodies. Samples were mounted with VECTASHIELD antifade mounting medium (H-1000-NB, Novios) and confocal microscopy was used for observation.

### Scanning electron microscopy

Mice were perfused with 1 × PBS and temporal bones were fixed in 2.5% glutaraldehyde at 4 °C overnight. After rinsing three times with 1 × PBS, the temporal bones were decalcified with 0.5 M EDTA at room temperature for 1-2 h, and the organ of Corti was dissected into apical, middle, and basal regions. Specimens were then rinsed in 1 × PB three times and post-fixed with 1% osmium tetroxide at room temperature for 1 h, after complete rinse with 1 × PB three times, the specimens were treated with 2% tannic acid (V900190-100G, Sigma). Specimens were rinsed with 1XPB and dehydrated through a graded ethanol series for 10 min at each gradient: 30%, 50%, 70%, 90%, and 100% at 4 °C. After dehydration, samples were critical point dried (Leica EM CPD300) and coated with 8 nm gold (Leica EM ACE200 Vacuum Coater) and observed with Aquilos Cryo-FIB (Thermo Scientific).

### Statistical analysis

All data are shown as the mean or mean ± SEM and all experiments were repeated at least three times. Statistical analyses were carried out using the Excel (Microsoft) and GraphPad Prism 8.0 software. ABR results were analyzed with a two-tailed, unpaired Student’s *t* tests. A value of *p* < 0.05 was considered statistically significant. Error bars and *n* values are defined in the respective figures and legends.

## Supplementary information

Supplementary figures and tables

## Data Availability

The datasets used and/or analyzed during the current study are available from the corresponding authors upon reasonable request.
