# Supplementary material for: AAV-ie-K558R mediated cochlear gene therapy and hair cell regeneration
Source: Signal Transduct Target Ther. 2022 Apr 22;7:109. doi: 10.1038/s41392-022-00938-8 (PMC9023545; doi:10.1038/s41392-022-00938-8)
Supplement: Supplementary file 1 — Supplementary figures and tables [file 41392_2022_938_MOESM1_ESM.docx]

**AAV-ie-K558R mediated cochlear gene therapy and hair cell regeneration**

Yong Tao^#1,2,3^, Xiaoyi Liu^#4,5^, Liu Yang^#,4,5^, Cenfeng Chu^4,5^, Fangzhi Tan^4,5^, Zehua Yu^4,5^, Junzi Ke^4,5^, Xiang Li^1,2,3^, Xiaofei Zheng^1,2,3^, Xingle Zhao^1,2,3^, Jieyu Qi^6,7^, Chao-Po Lin^5^, Renjie Chai*^6,7,8,9^, Guisheng Zhong*^1,4,5^, Hao Wu*^1,2,3^

1. Department of Otolaryngology-Head and Neck Surgery, Shanghai Ninth People’s Hospital, Shanghai Jiao Tong University School of Medicine, Shanghai 200011, P.R. China;
2. Ear Institute, Shanghai Jiao Tong University School of Medicine, Shanghai 200011, P.R. China;
3. Shanghai Key Laboratory of Translational Medicine on Ear and Nose Diseases, Shanghai 200011, P.R. China.

4. iHuman Institute, ShanghaiTech University, 201210, Shanghai, China. 5. School of Life Science and Technology, ShanghaiTech University, 201210, Shanghai, China.

6. State Key Laboratory of Bioelectronics, School of Life Sciences and Technology, Jiangsu Province High-Tech Key Laboratory for Bio-Medical Research，Southeast University, Nanjing 210096, China.
7. Co-Innovation Center of Neuroregeneration, Nantong University, Nantong 226001, China.
8. Institute for Stem Cell and Regeneration, Chinese Academy of Science, Beijing, China.
9. Beijing Key Laboratory of Neural Regeneration and Repair, Capital Medical University, Beijing, 100069, China

**This PDF file includes:**

Supplementary Figure 1-7

Supplementary Table 1


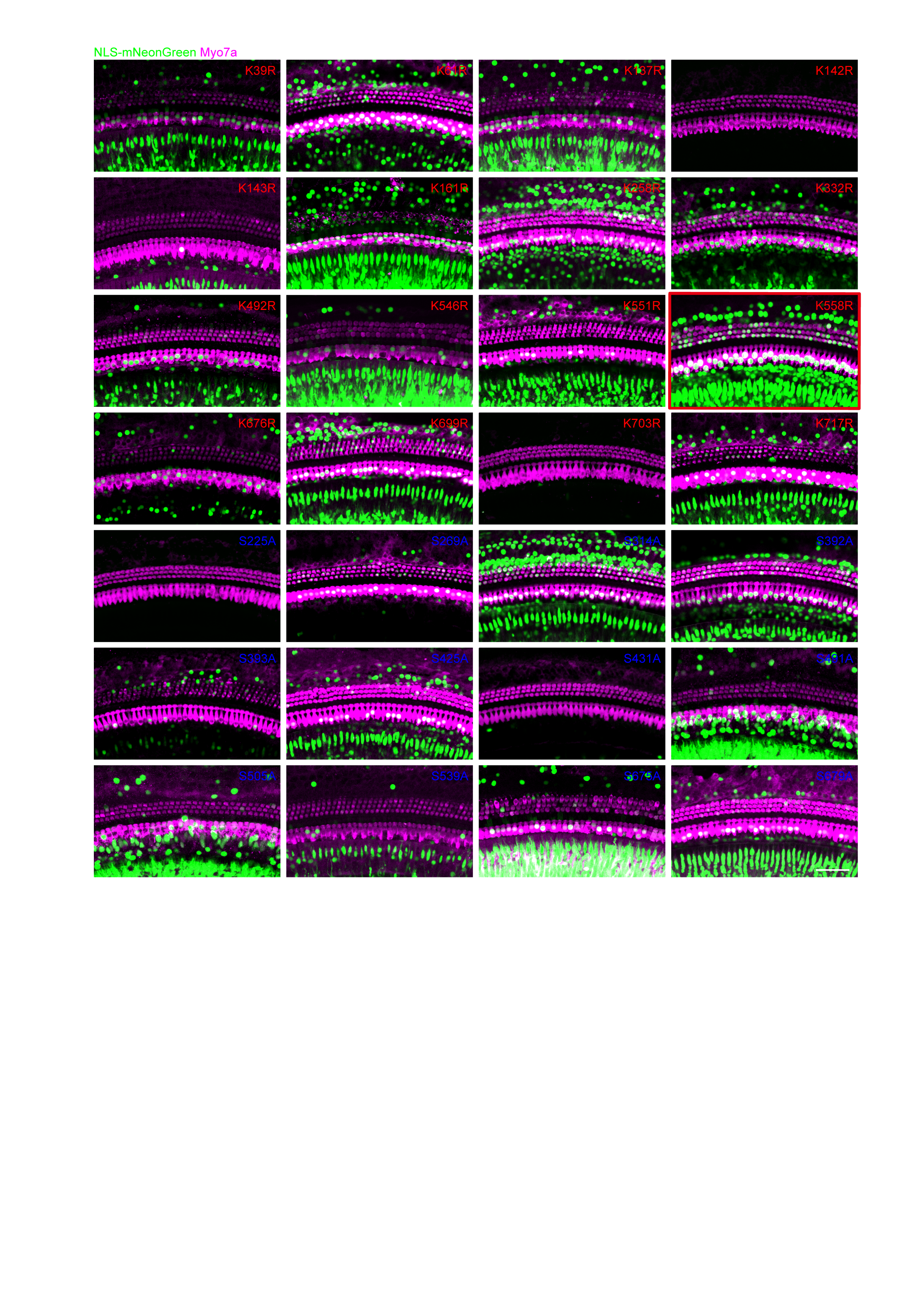


**Supplementary Figure 1. Transduce efficiency of AAV-ie variants.** Immunofluorescence images of AAV-ie S/K mutant vectors transduced cochlea, hair cell layer. All cochleae were harvested at P14 after microinjection with 1.5 μl of AAV stock solution at P3 and stained with anti-Myo7a antibody (magenta) and imaged for NLS-mNeonGreen fluorescence (green). Scale bar: 50 μm.


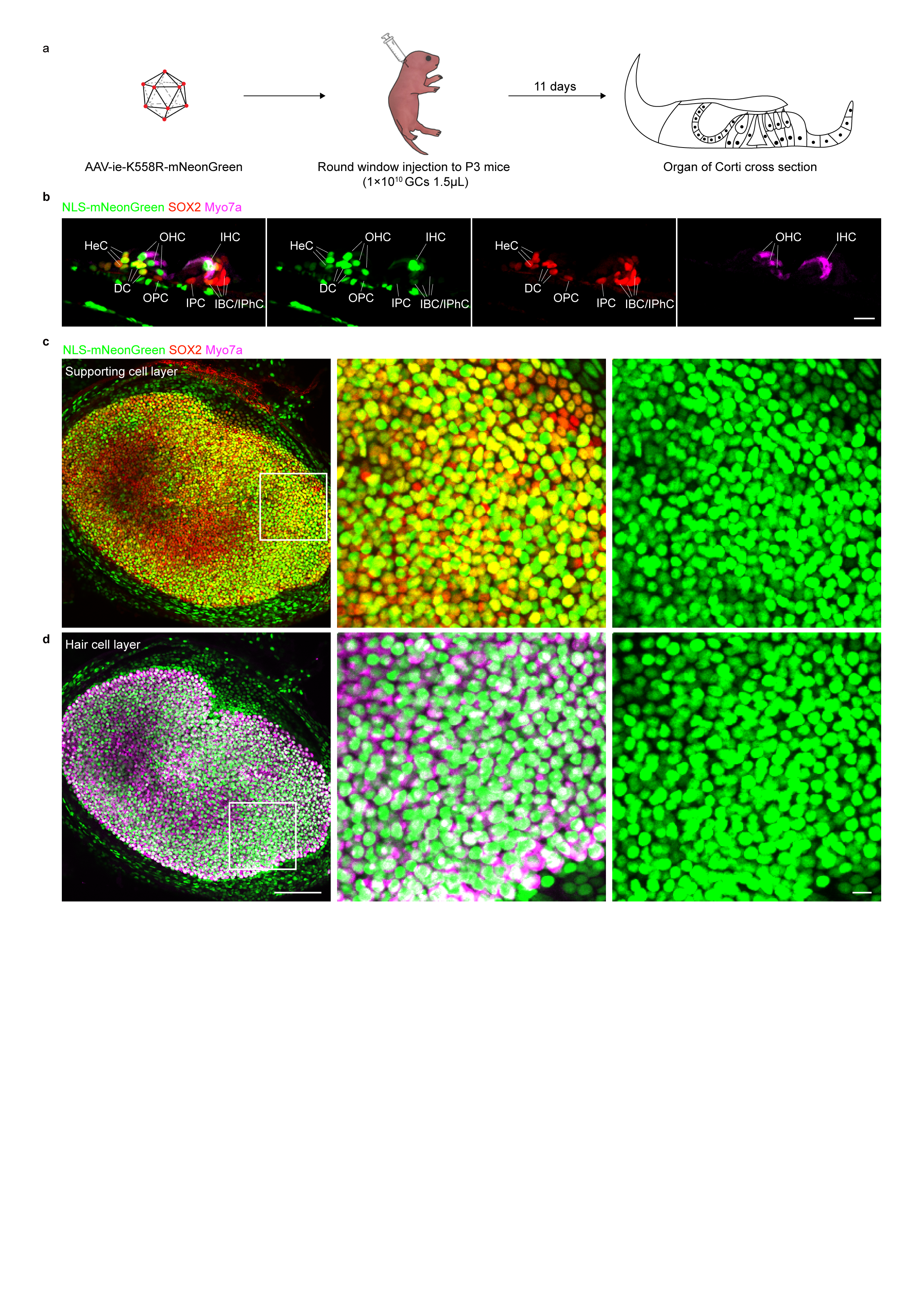


**Supplementary Figure 2. AAV-ie-K558R broadly transduces mouse cochlea, vestibular sensory epithelia.** **(a)** Schematic experimental setup of AAV-ie-K558R transduction in other cell types. P3 WT mouse were injected with AAV-ie-K558R-NLS-mNeonGreen at a dose of 1×10^10^ GCs. Tissues were harvested 11 days after injection. **(b)** Cryo-section of cochlea injected with AAV-ie-K558R- NLS-mNeonGreen and stained with antibodies against SOX2 (red) and Myo7a (magenta) and imaged for NLS-mNeonGreen fluorescence (green). The figure shows AAV-ie-K558R efficiently transduces cochlea hair cells and various types of supporting cells. Scale bar: 20 µm. **(c)** Left, representative confocal image of stained mouse utricular supporting cells. Right, magnified regions of boxed region in left image. **(d)** As in c, except for the utricular hair cell layer. Scale bars (c and d): left, 100 µm, right: 10 µm.


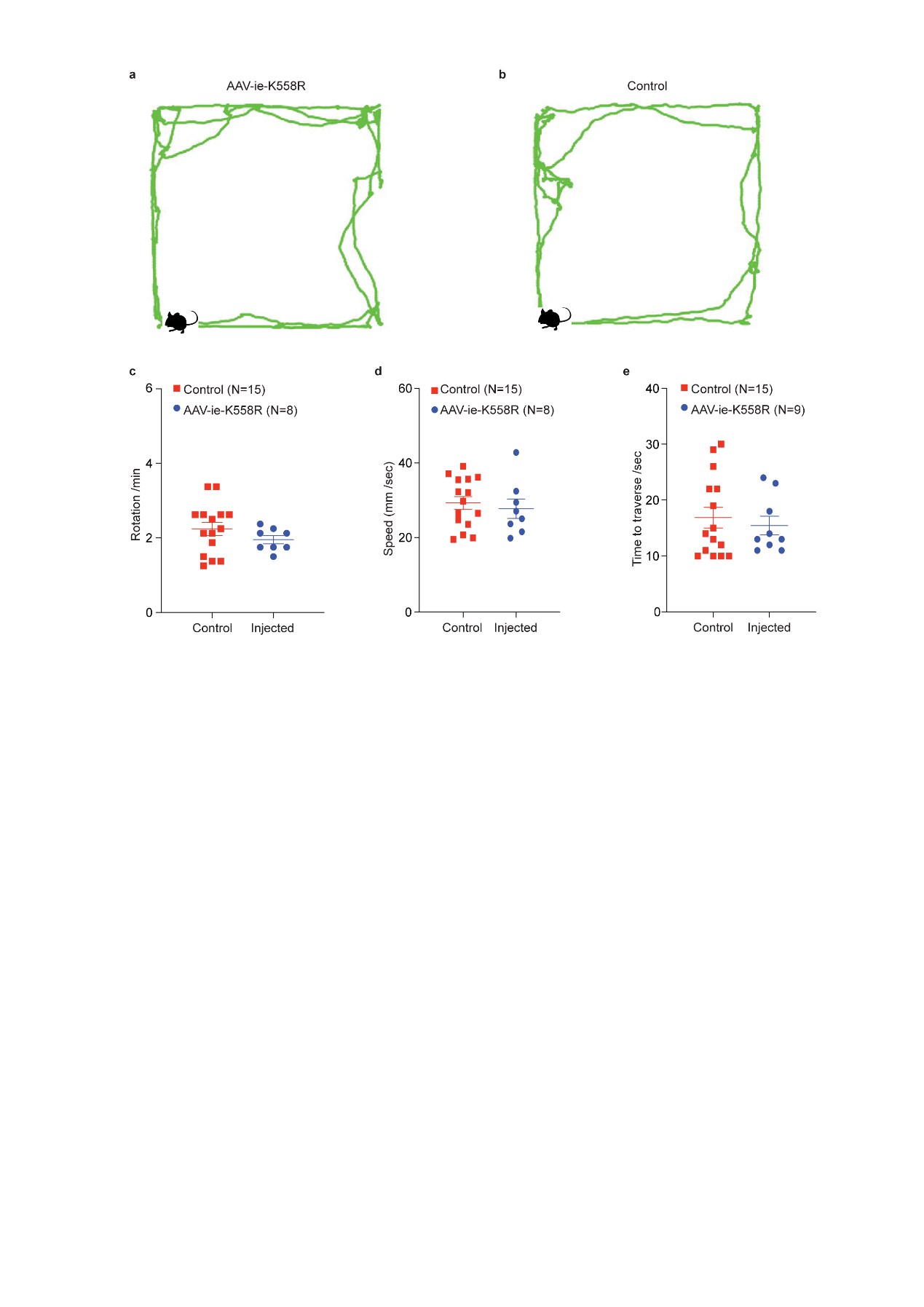


**Supplementary Figure 3. AAV-ie-K558R does not affect the vestibular function. (a)** Representative open–field pathway trace (120 s) from a P28 mouse injected with AAV-ie-K558R at P2.  **(b) Same as a, but** without injection. **(c)** Quantitation of the number of rotations per minute. **(d)** Quantitation of the speed per sec. **(e)** Quantitation of latency to traverse 80 cm narrow beam in AAV-ie-K558R injected and control mice. Error bars represent SEM, the number (n) of animals analyzed is indicated within the individual bars.

**
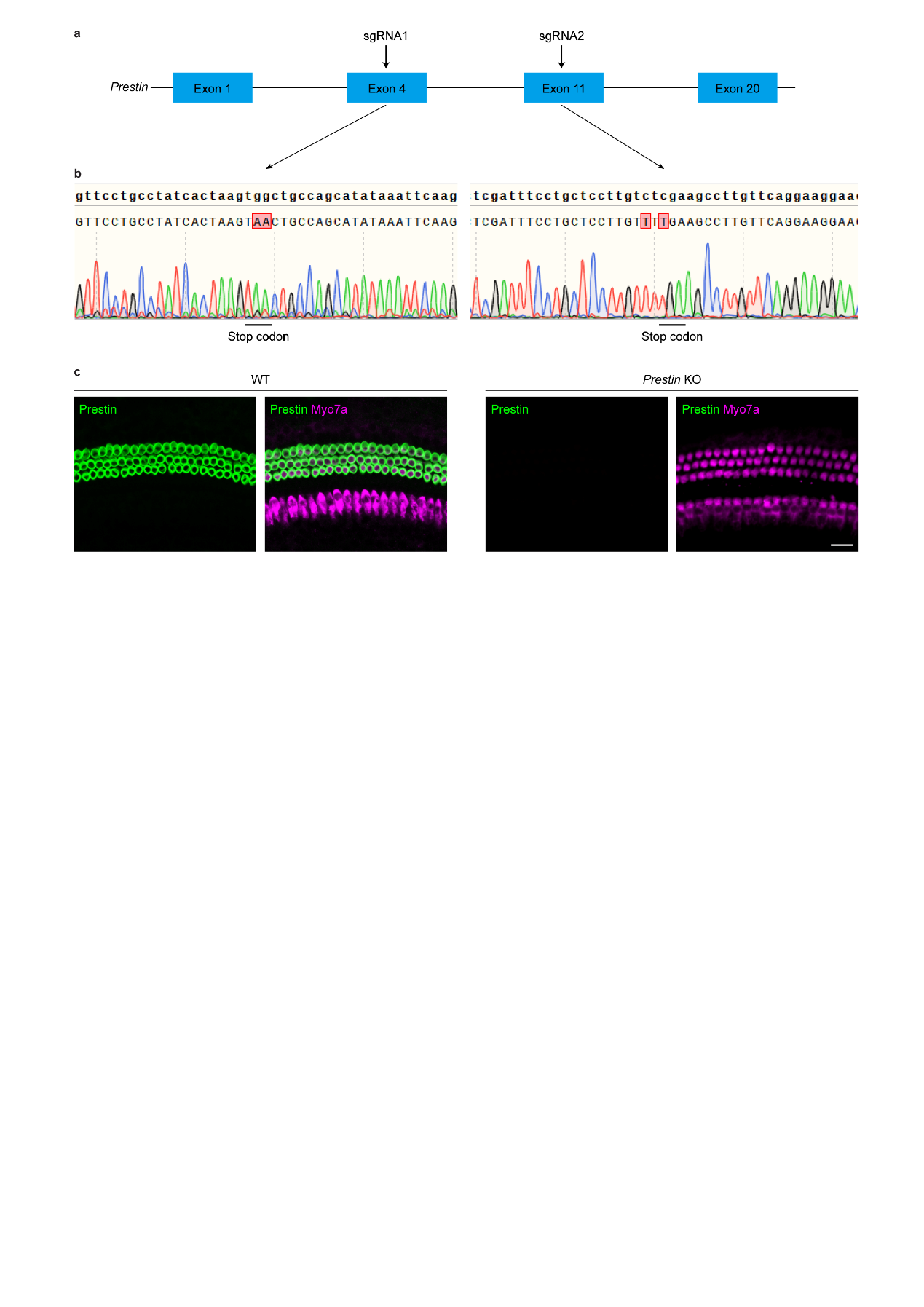
**

**Supplementary Figure 4. Generation and validation of *prestin* KO mouse line. (a)** *Prestin* KO mouse line was constructed via CRISPR guided base substitution. Two stop codons were simultaneously introduced to *Prestin* exon 4 and exon 11 coding sequence to cause an early transcribe termination. **(b)** Genotype of *Prestin* KO mice were validated by PCR amplification around mutate sites via primers described in methods and tested for sequencing. **(c)** Immunohistochemically lable of Prestin by specific antibody against Prestin(green) in WT mouse and *Prestin* KO mice clearly shows there is no Prestin expression in the OHCs of *Prestin* KO mice. Scale bar: 20 µm.


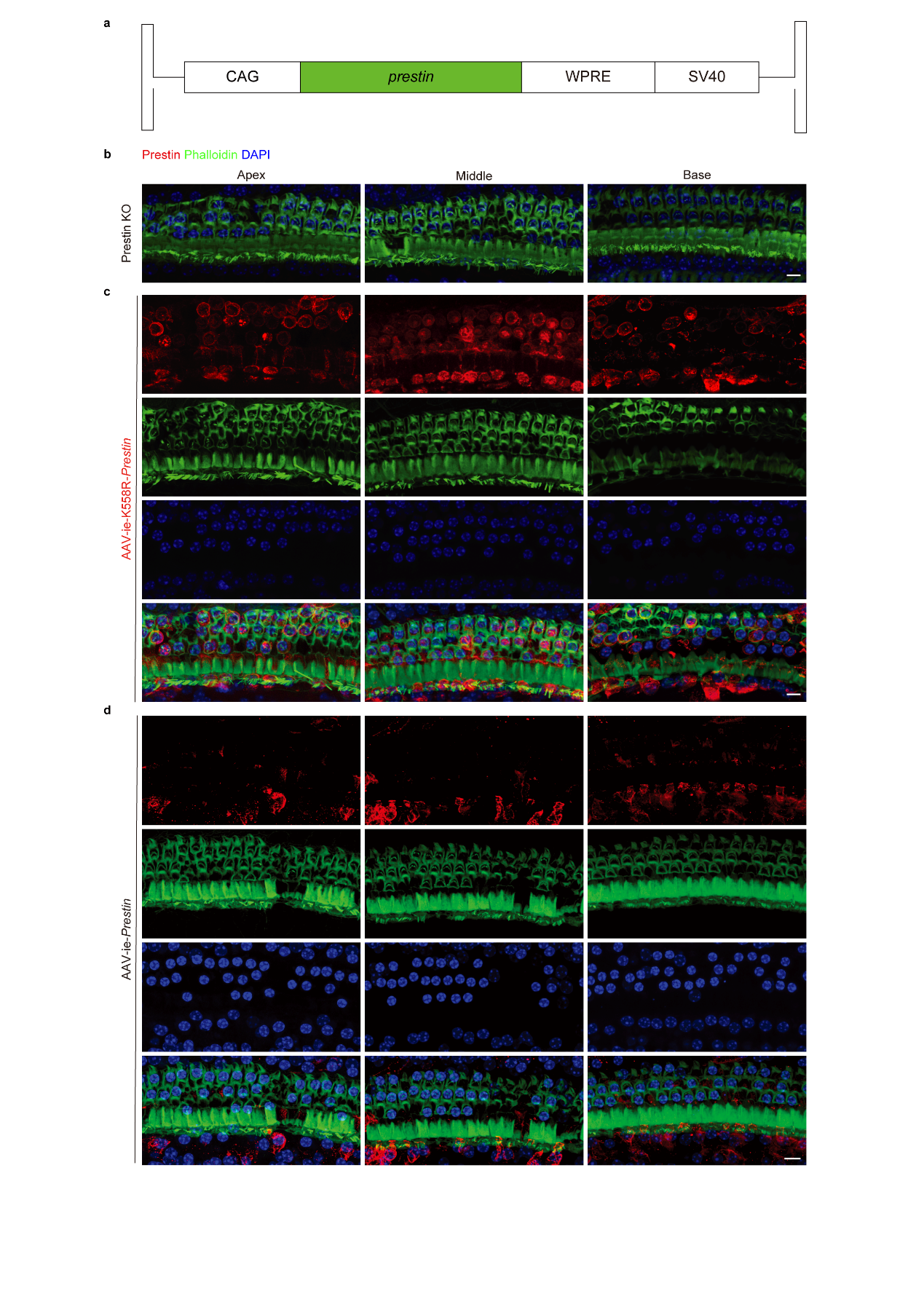


**Supplementary Figure 5. AAV-ie-K558R-prestin enables the expression of Prestin in both OHCs and IHCs. (a)** AAV-ie-K558R was used to package a single-stranded(ss) AAV genome that expresses prestin from the constitutive CAG promoter. **(b)** Phalloidin (green) was used to label F-actin and the morphology of HCs. Dapi (blue) was used to label cellular nuclei. *Prestin* KO mice did not express Prestin. Scale bar: 10 µm. **(c)** AAV-ie-K558R-*Prestin* enables the expression of Prestin in HCs and other types of cells. Scale bar: 10 µm. **(d)** AAV-ie-*Prestin* induces the expression of Prestin in HCs and other types of cells, but to a lesser extent. Scale bar: 10 µm.


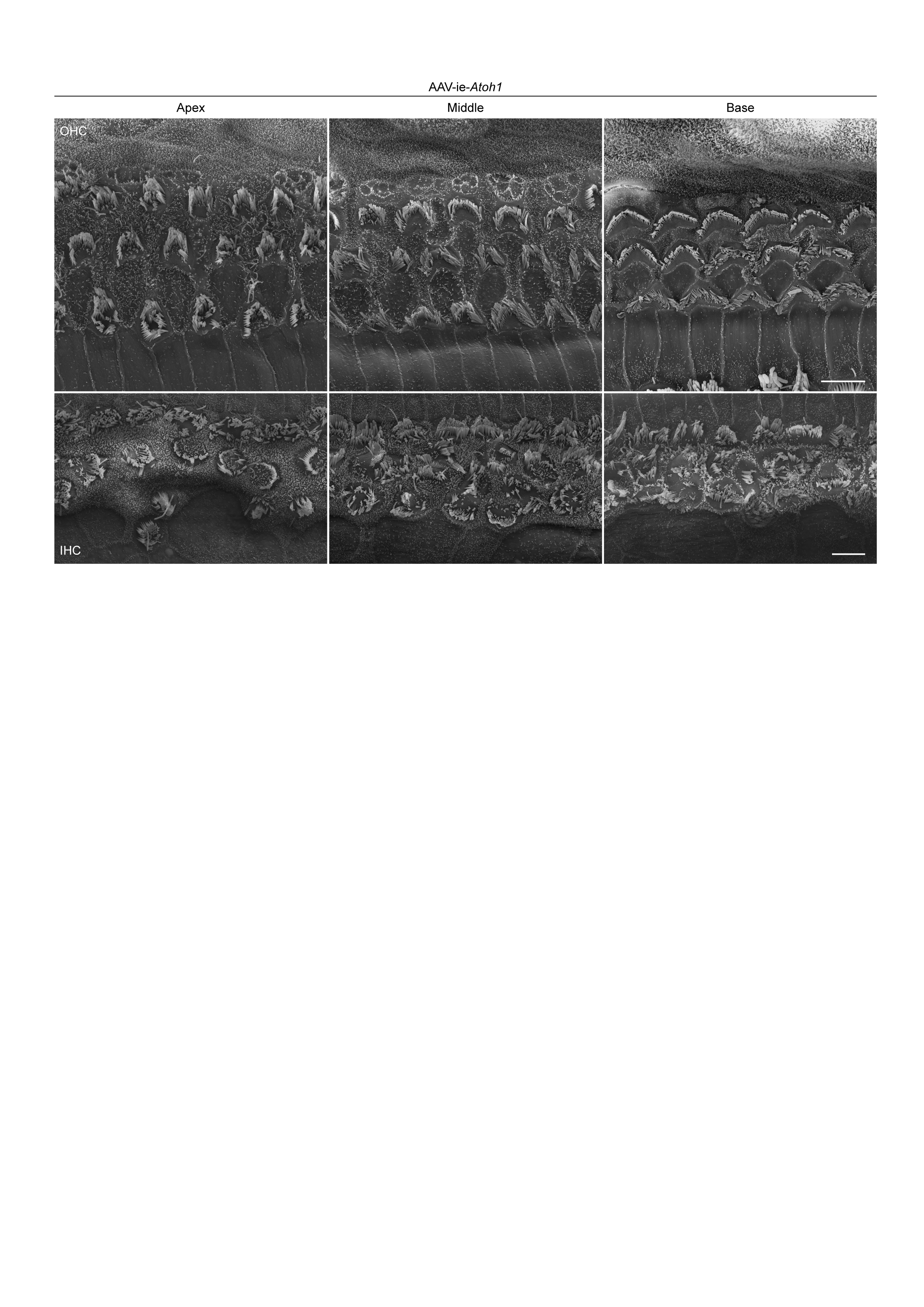


**Supplementary Figure 6. AAV-ie-*Atoh1* induced HC-like cells in neonatal mice.** SEM images of cochlea injected with AAV-ie -*Atoh1* (1×10^10^ GCs) on P14 in apical, middle and basal region. Scale bar: 10 µm.


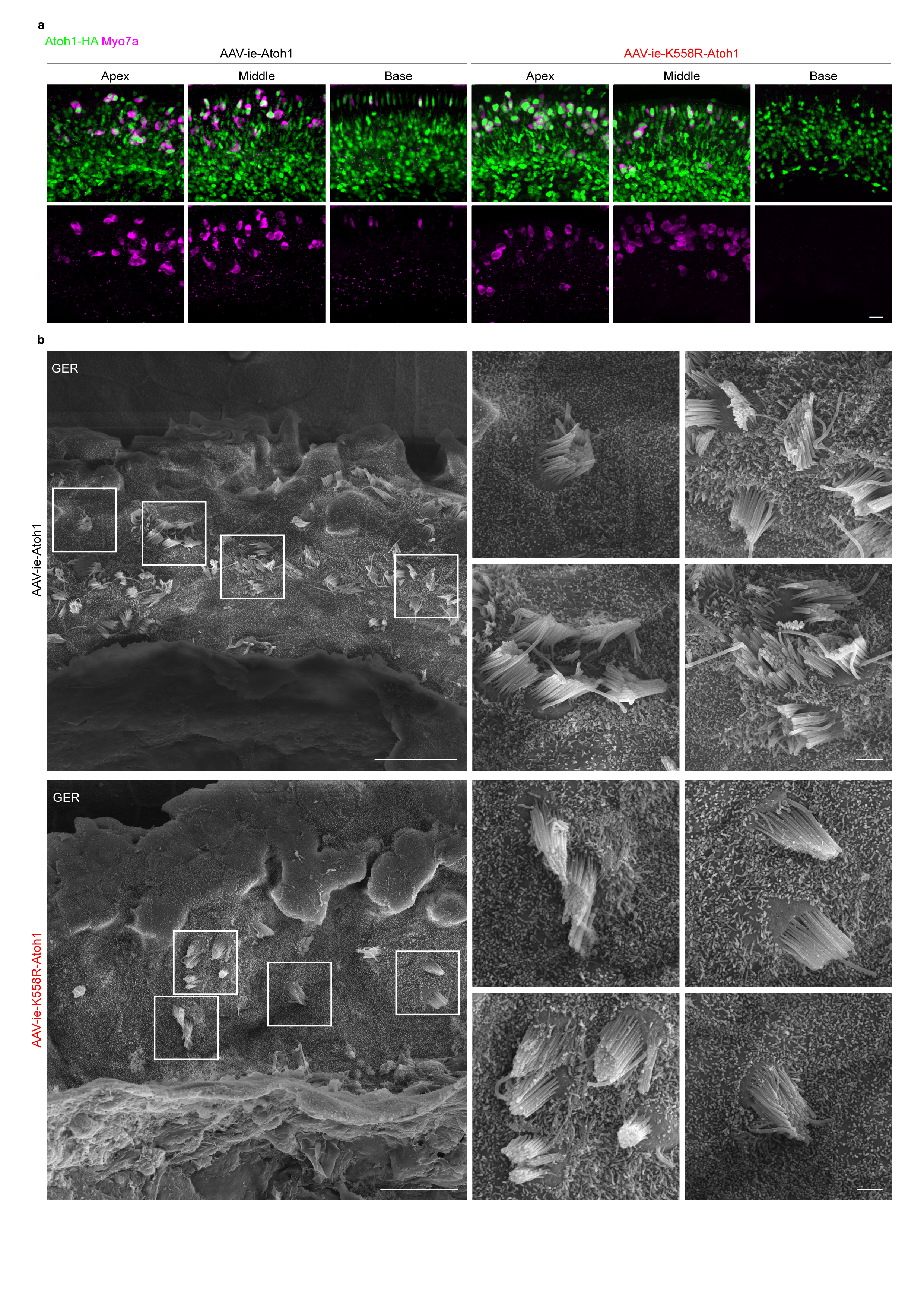


**Supplementary Figure 7. AAV-ie-K558R-*Atoh1* induces HC-like cells in the GER region. (a)** Immunofluorescence imaging of cochlea GER transduced with AAV-ie-*Atoh1* and AAV-ie-K558R-*Atoh1* at dose of 1×10^10^ GCs. Both AAV-ie-*Atoh1* and AAV-ie-K558R-*Atoh1* can generate Myo7a positive cells in the GER region. Scale bar: 5 µm. **(b)** SEM images of cochlea injected with AAV-ie -Atoh1 and AAV-ie-K558R-Atoh1 on P14 in GER region. SEM images verified vector-induced HC-like cell regeneration that developed cilium bundles. Scale bar: left, 20 µm, right, 1µm.

| **Mutants** | **Mutate primers (5’-3’)** |
| --- | --- |
| K39R | CCACCACCAAAGCCCGCAGAGCGGCA**TCG**GGACGACGCC  Lys→Arg |
| K61R | CCCTTCAACGGACTCGAC**AGG**GGAGAGCCGGTCAACGAGG  Lys→Arg |
| K137R | TCTTGGTCTGGTTGAGGAAGCGGCT**CGG**ACGGCTCCTGGAA  Lys→Arg |
| K142R | GCTAAGACGGCTCCTGGA**AGG**AAGAGGCCTGTAGAGCAC  Lys→Arg |
| K143R | GGCTCCTGGAAAG**CGG**AGGCCTGTAGAGCACTCTCCTGTGG  Lys→Arg |
| K161R | ACTCCTCCTCGGGAACCGGA**CGG**GCGGGCCAGCAGCCTGCA  Lys→Arg |
| K258R | CAACAACCACCTCTAC**CGG**CAAATCTCCAACAGCACATC  Lys→Arg |
| K332R | CACGCAGAATGAAGGCACC**AGG**ACCATCGCCAATAACCTC  Lys→Arg |
| K492R | CAGCGAGTATCA**CGG**ACATCTGCGGATAACAACAACAGTGAAT  Lys→Arg |
| K546R | CGGGGTTCTCATCTTTGGG**CGG**CAAGGCTCAGAGAAAACAAAT  Lys→Arg |
| K551R | GGGAAGCAAGGCTCAGAG**AGA**ACAAATGTGGACATTG  Lys→Arg |
| K558R | GAGAAAACAAATGTGGACATTGAA**CGG**GTCATGATTACAGACGA  Lys→Arg |
| K676R | CCTTCAACCAGTCA**CGG**CTGAACTCTTTCATCACCCAGTATTCTA  Lys→Arg |
| K699R | GATCGAGTGGGAGCTGCAG**AGG**GAAAACAGCAAGCGCTGG  Lys→Arg |
| K703R | CTGCAGAAGGAAAACAGC**AGG**CGCTGGAACCCCGAGATCC  Lys→Arg |
| K717R | GTACACCTCCAACTACTAC**AGA**TCTACAAGTGTGGACTTTG  Lys→Arg |
| S225A | GGAGTGGGTAATTCC**GCG**GGAAATTGGCATTGCGATTCC  Ser→Ala |
| S269A | GCACATCTGGAGGATCT**GCA**AATGACAACGCCTACTTCGG  Ser→Ala |
| S314A | CCGGCCCAAGAGACTC**GCC**TTCAAGCTCTTCAACATCCAG  Ser→Ala |
| S392A | CAGGCCGTGGGACGC**GCC**TCCTTCTACTGCCTGGAATAC  Ser→Ala |
| S393A | GTAGTCAGGCCGTGGGACGCTCC**GCC**TTCTACTGCCTGG  Ser→Ala |
| S425A | GACGTGCCTTTCCACAGC**GCC**TACGCCCACAGCCAGAGC  Ser→Ala |
| S431A | CTACGCCCACAGCCAG**GCC**TTGGACCGGCTGATGAATCC  Ser→Ala |
| S491A | CGCCAGCAGCGAGTA**GCA**AAGACATCTGCGGATAACAACAAC  Ser→Ala |
| S505A | GATAACAACAAC**GCT**GAATACTCGTGGACTGGAGCTACCCGGT  Ser→Ala |
| S539A | GTTTTTTCCTCAG**GCC**GGGGTTCTCATCTTTGGGAAGCAAGGC  Ser→Ala |
| S675A | ATCCTCCGACCACCTTCAACCAG**GCA**AAGCTGAACTCTTTCATC  Ser→Ala |
| S679A | CAAAGCTGAAC**GCT**TTCATCACCCAGTATTCTACTGGCCAAGTCA  Ser→Ala |

**Supplementary Table 1. Sequences of AAV-ie capsid amino acid mutations.** Capital letters represent the codons encoding amino acids, and red letters represent the bases after mutation.
